# Probing the Interaction of Brain Fatty Acid Binding Protein (B-FABP) with Model Membranes

**DOI:** 10.1371/journal.pone.0060198

**Published:** 2013-03-28

**Authors:** Fábio Dyszy, Andressa P. A. Pinto, Ana P. U. Araújo, Antonio J. Costa-Filho

**Affiliations:** 1 Grupo de Biofísica Molecular Sérgio Mascarenhas, Instituto de Física de São Carlos, Universidade de São Paulo, São Carlos, Sao Paulo, Brazil; 2 Laboratório de Biofísica Molecular, Departamento de Física, Faculdade de Filosofia, Ciências e Letras de Ribeirão Preto, Universidade de São Paulo, Ribeirão Preto, Sao Paulo, Brazil; Universidad de Granada, Spain

## Abstract

Brain fatty acid-binding protein (B-FABP) interacts with biological membranes and delivers polyunsaturated fatty acids (FAs) via a collisional mechanism. The binding of FAs in the protein and the interaction with membranes involve a motif called “portal region”, formed by two small α-helices, A1 and A2, connected by a loop. We used a combination of site-directed mutagenesis and electron spin resonance to probe the changes in the protein and in the membrane model induced by their interaction. Spin labeled B-FABP mutants and lipidic spin probes incorporated into a membrane model confirmed that B-FABP interacts with micelles through the portal region and led to structural changes in the protein as well in the micelles. These changes were greater in the presence of LPG when compared to the LPC models. ESR spectra of B-FABP labeled mutants showed the presence of two groups of residues that responded to the presence of micelles in opposite ways. In the presence of lysophospholipids, group I of residues, whose side chains point outwards from the contact region between the helices, had their mobility decreased in an environment of lower polarity when compared to the same residues in solution. The second group, composed by residues with side chains situated at the interface between the α-helices, experienced an increase in mobility in the presence of the model membranes. These modifications in the ESR spectra of B-FABP mutants are compatible with a less ordered structure of the portal region inner residues (group II) that is likely to facilitate the delivery of FAs to target membranes. On the other hand, residues in group I and micelle components have their mobilities decreased probably as a result of the formation of a collisional complex. Our results bring new insights for the understanding of the gating and delivery mechanisms of FABPs.

## Introduction

Fatty acid binding proteins (FABPs) comprise a family of small cytoplasmic proteins (14–16 kDa) with specific patterns of tissue distribution and development-dependent expression [1]. The main function of FABPs is the reversible binding and transport of hydrophobic ligands, including long-chain, saturated or unsaturated fatty acids (FAs), which are part of a complex balance of processes such as energy storage, gene expression, cell growth, membrane synthesis, inflammatory and metabolic responses, activity of ion channels and receptors, and modifications of the structure of proteins and membranes [2]–[5]. FABPs are also able to interact with peroxisome proliferator-activated receptor (PPAR) and to regulate their gene expression [6]–[9]. In humans, nine members of the FABP family have been identified, which are expressed in organs and tissues involved in lipid metabolism [10]. The family is subdivided into four groups according to their homology and to their affinity for ligands [11].

The similarity in primary structure varies considerably among the members of FABP families, ranging from 28 to 70% [12]. However, the tertiary structures are highly conserved [13]–[15], consisting of 10 anti-parallel β-strands, arranged in two nearly orthogonal sets, forming a 500 Å^3^ β-barrel that accommodates the FA molecule. Furthermore, FABPs present two small α-helices, A1 and A2, connected by a loop, forming a portal region. This structure modulates the entry of FAs in the cavity and is believed to be responsible for the interaction between FABP and membranes [16].

The gene for human B-FABP was isolated and sequenced in 1997 [17], showing that human B-FABP has 131 amino acids, molecular mass of 14.8 kDa, and pI of 5.4. Besides being found in the brain, B-FABP is also encoded in glial cells in the retina and mammary glands [10]. B-FABP has high affinity for polyunsaturated FAs n-3, such as eicosapentaenoic acid (20∶5) and docosahexaenoic acid (22∶6, DHA), which are present in high concentrations in the plasma membrane of nervous tissue cells [13]. A deficiency of this type of FA affects the behavior of rats, leading to learning disabilities and to changes in electrophysiological visual activities [18]–[21]. B-FABP also interacts with monounsaturated n-9 FAs, such as palmitoleic acid (16∶1) and oleic acid (18∶1, AO), with dissociation constants of the same order of those found for polyunsaturated n-3 fatty acids [13]. The FA carboxilate moiety interacts with side chains of residues Arg^106^, Arg^126^ and Tyr^128^, and a complex network of van der Waals interactions involving about 20 amino acids stabilize the hydrocarbon chain of the FA [13].

During the early stage of embryonic development, there is high expression of B-FABP in the whole brain, but expression levels decrease noticeably with the progress of neuronal differentiation [22]. In patients with Down syndrome [23] and schizophrenia [24], B-FABP is super expressed. Interestingly, mice deficient in B-FABP proved to be viable without macroscopic abnormalities, but showed altered behavior, such as increased anxiety and fear, without changes neither in memory nor in learning ability [24], [25]. Studies also indicate that B-FABP influences the correct migration of neurons to the cerebral cortex [22] and a decrease in tumor growth in models of murine breast cancer occurs when B-FABP is overexpressed [26], [27]. B-FABP has been suggested as a possible tumor marker in renal cell carcinomas and neuroblastomas [28]–[31].

It has been proposed that the delivery of lipids and FAs by proteins of the FABP family to their final destination occurs through two mechanisms: diffusional or collisional [32], [33]. In the diffusional-dependent mechanism, the FA molecule is released from its binding site when the protein is near the acceptor membrane and the FA diffuses in the aqueous phase. There are studies showing that the nature of the vesicle lipid composition influences the diffusional-dependent mechanism [34] and suggest that the membrane-water interface is responsible for inducing protein conformational changes with subsequent release of the FA molecule [35], [36].

In the collisional mechanism, it is believed that the net positive charge of the portal region directs the formation of a collisional complex with the membrane [37]–[39]. The helix-turn-helix motif that forms the portal would then be responsible for the gating and the delivery of the FA to the target membrane [16], [40]. FABPs whose portal region has a negative charge, such as liver FABP [41], does not seem to rely on a collision to deliver FAs [42], [43]. This points to the relevance of Lys residues as well as amphipaticity of the helices located in the portal for the binding and delivery mechanisms. It has also been shown that the collisional mechanism is, as expected, highly dependent on both the concentration of vesicles and on the lipid composition of the acceptor vesicles. Anionic lipids increased by 15 times the throughput of the fluorescent FA from the protein to the acceptor vesicles [33]. In particular, Thumser *et al.* [44] showed that B-FABP performs the delivery of FAs by collisional mechanism. However, there is no structural information for B-FABP concerning which residues participate in protein-membrane interaction or a direct evidence of B-FABP-membrane interaction.

Electron spin resonance (ESR) along with site-directed spin labeling (SDSL) has been used to investigate structure and dynamics [45]–[51], to measure distances [52] and to monitor changes in mobility or solvent accessibility in proteins [53]–[56]. In all these papers, the structural and dynamical information is reported by the so-called R1 side-chain, which is obtained from the selective labeling of the side chain of a cysteine residue with the probe (1-oxyl-2,2,5,5-tetramethyl-Δ3-pyrroline-3-methyl) methanethiosulfonate (MTSSL). In the present paper, we applied ESR to site-directed spin labeled mutants of B-FABP and to spin labeled stearic acid molecules in order to probe the changes in the protein as well as in the membrane model induced by the interaction between them. We found alterations for both protein and membrane models: (1) the helices composing the portal region of B-FABP underwent a conformational change so that side chains of residues pointing outward to the solvent were less mobile in the presence of the membrane mimetic, whereas residues whose side chains point inward assumed a less ordered state that increases the inner volume between the helices, thus possibly allowing the delivery of FA molecule; (2) the probes in the membrane model showed a decrease in mobility induced by B-FABP, which is compatible with the stabilization observed for the protein residues with side chains oriented outward from the portal interior.

## Materials and Methods

### Materials

BL21(DE3) cells and pET28 expression vector were purchased from Novagen (Milwaukee, WI, USA). pETSUMO expression system were purchased from Life Technologies (Carlsbad, CA, USA). Lysophospholipids LPC (1-palmitoyl-2-hydroxy-*sn*-glycero-3-phosphocholine) and LPG (1-palmitoyl-2-hydroxy-*sn*-glycero-3-phospho-(1′-rac-glycerol)) were purchased from Avanti Lipids Inc. (Alabaster, AL, USA). Spin probe (1-oxyl-2,2,5,5-tetramethyl-Δ3-pyrroline-3-methyl) methanethiosulfonate (MTSSL) were obtained from Toronto Research Chemicals (Toronto, Ontario, Canada). Probes *n*-(4,4-dimethyloxazolidine-N-oxyl)stearic acid, (*n*-SASL, where n = 5, 12 or 16) and other reagents were purchased from Sigma-Aldrich (St. Louis, MO, USA) and used without further purification.

### Cloning, expression and purification of B-FABP and their mutants

B-FABP mutants containing single cysteine substitutions were generated using the QuickChange method (Stratagene, La Jolla, CA, USA). The genes of each mutant were sequenced in a 3130 Genetic Analyzer (Applied Biosystems, Foster City, CA, USA). B-FABP wild-type and mutants R30C, Q31C, V32C and N34C were transformed into *Escherichia coli* BL21(DE3) cells using pET28 plasmids. Cell cultures were grown to an O.D. = 1.0, and the protein expression was induced overnight by adding 0.3 mM (final concentration) of isopropyl β-D-1-thiogalactopyranoside (IPTG). The cells were harvested by centrifugation. Purification was performed using an adapted protocol from previous works [57], [58]. Briefly, cell pellets were ressuspended in 20 mM phosphate buffer (pH 8.0), containing 0.1 M NaCl, 8 M urea, 1 mM dithiothreitol (DTT) and sonicated (Fischer Scientific, Waltham, MA, USA) in ice bath ten times for 30 seconds at 10% power with an intermittent 45 seconds interval to allow for sample cooling. The sample was then left in ice bath for 15 minutes. Cell debris was pelleted by centrifugation at 12,000 rpm for 30 minutes. The supernatant was diluted 10 times in 20 mM phosphate buffer (pH 8.0) containing 0.1 M NaCl, 1M arginine, 1 mM DTT, and left at 4°C for 1 hour under gentle stirring. The sample was submitted to a 12-hour dialysis (membrane MWCO 8000 Da) against 20 mM phosphate buffer (pH 8.0), containing 0.1 M NaCl, 1 mM DTT, changed one time 6 hours after the start of dialysis. The sample was loaded in a 4 mL Ni-NTA column (Qiagen, Hilden, Germany) equilibrated with 20 mM phosphate buffer (pH 8.0), containing 0.1 M NaCl, 1 mM DTT. B-FABP was eluted with a linear gradient from 0 to 200 mM imidazol. His-tag cleavage was performed by thrombin and then passing the sample through benzamidine and Ni-NTA columns in order to remove thrombin and residual His-tagged B-FABP.

B-FABP mutants D17C, E18C, M20C, K21C and G33C were prepared similarly to the previous ones, except for the transformation into *Escherichia coli* BL21(DE3) cells using the pETSUMO plasmids. B-FABP mutants were cleaved by using SUMO protease and passing the sample through Ni-NTA columns to remove SUMO protease and residual SUMO-tagged B-FABP. After purification, these mutantswere submitted to denaturation in 20 mM phosphate buffer (pH 8.0), containing 0.1 M NaCl, 8 M urea, 1 mM DTT for 15 minutes in ice bath. Then the sample was left in ice bath for 15 minutes. The sample was diluted 10 times in 20 mM phosphate buffer (pH 8.0) containing 0.1 M NaCl, 1M arginine, 1 mM DTT, and left at 4°C for 1 hour with gentle shaking. After that the sample was submitted to a 12-hour dialysis (membrane MWCO 8000 Da) against 20 mM phosphate buffer (pH 8.0), containing 0.1 M NaCl, 1 mM DTT, changed one time 6 hours after the start of the dialysis.

When required, proteins were concentrated in an Amicon® Ultra-15 concentrator fitted with Ultracel-10 membrane (Merck Millipore, Billerica, MA, USA). Protein purity was assessed by using gel electrophoresis in a 15% SDS-PAGE system.

### Spin labeling of B-FABP mutants

B-FABP mutants purified as described above were passed through a 5 mL HiTrap Desalting column (GE Healthcare, Uppsala, Sweden) equilibrated with labeling buffer (20 mM phosphate, 0.1 M NaCl, pH 8.0), and immediately reacted with a 10-fold molar excess of MTSSL. Labeling reactions were allowed to proceed overnight at 4°C, under gentle stirring. Excess spin reagent was removed using the same desalting column, eluted with 20 mM phosphate, pH 8.0 buffer. Spin-labeled B-FABPmutants were concentrated to 50–100 µM in an Amicon® Ultra-15 concentrator fitted with Ultracel-10 membrane (Merck Millipore, Billerica, MA, USA).

### Preparation of labeled micelles

Micelles containing the probes *n*-SASL were obtained by evaporating volumes of 3 µL of stock solutions of *n*-SASL and of either LPC or LPG, with a subsequent period of 1 h in vacuum. After this step, the dry film containing the lysophospholipds and *n*-SASL probes (1 mol% final concentration) was resuspended in 100 µL of 20 mM phosphate buffer (pH 8.0), 1 mM DTT (control), and the same buffer containing G33C B-FABP. For all cases, the protein to lysophospholipid ratio was 1∶50 (mol∶mol).

### ESR measurements

Continuous wave ESR spectroscopy was carried out at room temperature (22±1°C) on a Varian E109 spectrometer operating at X-band. Solutions containing labeled B-FABP mutants in solution or in the presence of LPC or LPG micelles were mixed and drawn into capillary tubes for ESR experiments. Final protein concentration varied from 50 to 100 µM and the protein to lysophospholipid ratio was 1∶50 (mol∶mol). Acquisition conditions were: modulation amplitude, 1.0 G; modulation frequency, 100 kHz; field range, 160 G. Incident microwave power and number of scans were optimized to achieve the best signal-to-noise ratios for each case.

The B-FABP spectral analysis was performed according to empirical parameters described by Columbus and Hubbell [59]. For 5-SASL, the order parameter (S) [60] was measured from the spectra of the micelle-bound probe, as:
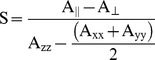
where 2A_‖‖_ is the maximum hyperfine splitting and 2A_⊥_ is the inner hyperfine splitting. A_xx_, A_yy_ and A_zz_ are the principal components of the hyperfine tensor and were taken as 6.0, 6.0 and 32.0 G, respectively [60]. For 16-SASL, correlation times were calculated by assuming pseudoisotropic motion based on the expression [60]:
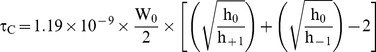
where W_0_ is the peak-to-peak width of the central line of resonance, and h_+1_, h_0_ and h_−1_ are the heights of the low field, central and high field lines, respectively.

When necessary, the spectrum of free label was subtracted using Multi-Component EPR Fitting v2, developed by Christian Altenbach (UCLA, Los Angeles, USA), and available at https://sites.google.com/site/altenbach/downloads.

### CD measurements

CD spectra were recorded in a JASCO J-815 (Jasco Inc., Maryland, USA) spectropolarimeter calibrated with (+)−10-camphorsulphonic acid. Measurements in the far-UV region (198–250 nm) were made using a 0.1 cm cell, under the following conditions: data pitch, 0.2 nm; scan speed, 50 nm/min; response, 1 s; band width, 1 nm. The protein concentration was 12 µM in 20 mM phosphate buffer (pH 8.0). CD values are reported as mean residue ellipticity [θ]_MRE_ [61].

## Results and Discussion

In this report, we studied the interactions between the *apo* form of the human brain fatty acid-binding protein (B-FABP) and zwitterionic (LPC) or anionic (LPG) micelles, making use of a combination of site-directed spin labelingand electron spin resonance (ESR). We produced nine mutants of B-FABP along α-helices A1 and A2 located in the region known as “portal region”. The portal region is recognized as being fundamental in FABP-membrane interaction, modulating both the delivery mechanism of fatty acids [16], [40], [62] and the protein-membrane interaction itself [16]. Thus, probes positioned in the α-helices are useful for analyzing the dynamics of the residues in the presence of biomimetic systems and their importance for the protein-membrane interactions. In addition, we studied changes from the perspective of the micelles as reported by labeled FAs 5-, 12- and 16-SASL.

**Figure 1:**
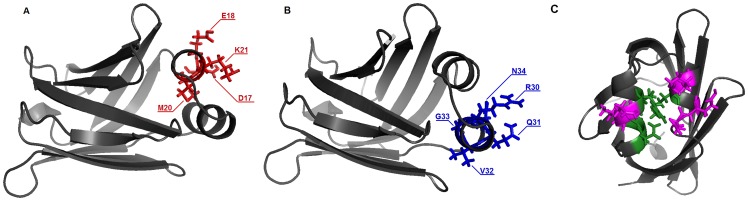
Top view of the three-dimensional structure of human B-FABP (PDB ID 1JJX), showing the native residues that were mutated to Cys residues in α-helix A1 (A) or in α-helix A2 (B). Panel (C): front view of the three-dimensional structure of human B-FABP (PDB ID 1JJX), showing the residues that form the group I (magenta), and the residues which form group II (green) discussed in the text.

**Figure 2:**
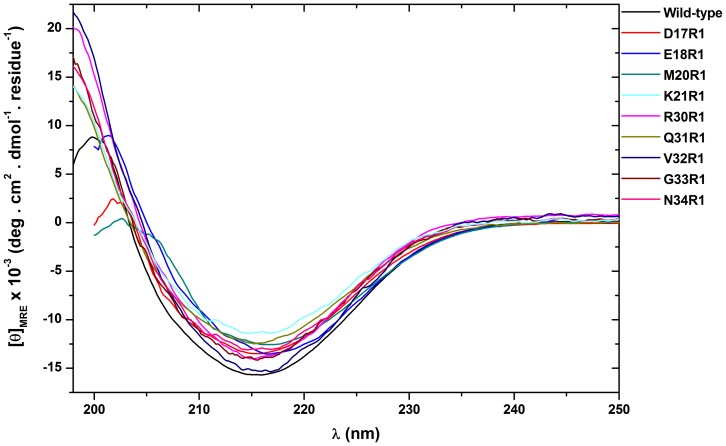
CD spectra of B-FABP wild type and their mutants in 20 mM phosphate buffer (pH 8.0). All spectra are characterized by a negative minimum around 215 nm, which is typical in β-rich proteins. The similarity of the spectra suggests that the introduction of the Cys residue in the mutants of B-FABP has not significantly altered their overall secondary structure arrangement.

B-FABP has in its structure two native Cys residues at positions 5 and 81. These residues were mutated to Ala and Ser, respectively, so that there is only one Cys residue in the structure, i.e. the Cys introduced by mutagenesis. Mutations in the following B-FABP residues were used: Asp^17^, Glu^18^, Met^20^ and Lys^21^ in α-helix A1, and Arg^30^, Gln^31^, Val^32^, Gly^33^ and Asn^34^ in α-helix A2 (Fig. 1). The mutations and the labeling process with the paramagnetic probe MTSSL does not seem to significantly alter the content of the secondary structure arrangement of the mutants when compared to the secondary structure of B-FABP wild type. Protein primary structure of FABP family presents variable similarity, ranging from 28 to 70%, but the tertiary structure is highly conserved [12]. Circular dichroism spectra (Fig. 2) are typical of β-sheet rich proteins, with a characteristic minimum at 215 nm [63], and are in agreement with the results obtained for I-FABP [64] and L-FABP [62].

**Figure 3:**
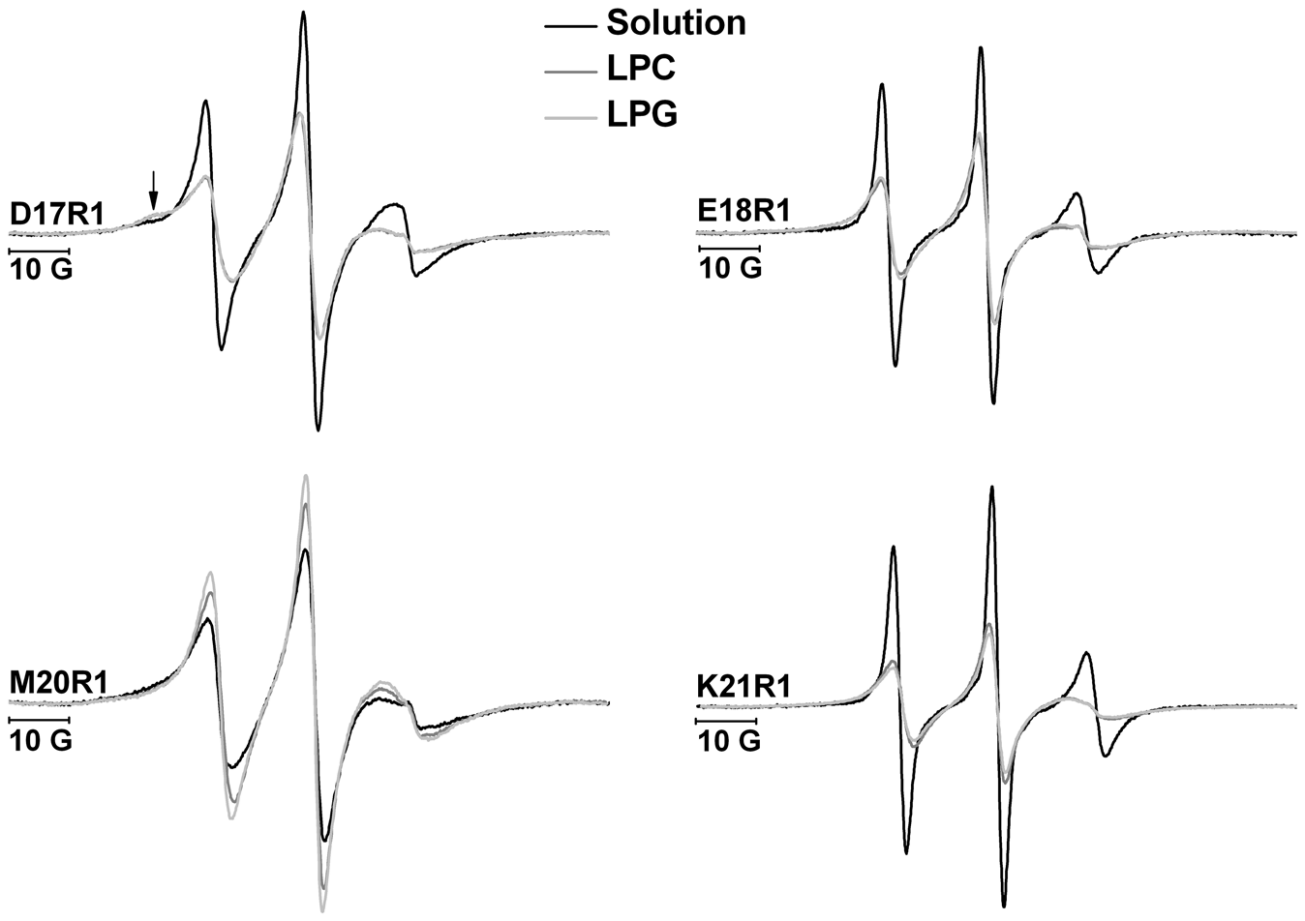
ESR spectra of B-FABP mutants D17R1, E18R1, M20R1 and K21R1, in the absence (black) and in the presence of LPC (gray) or LPG (light gray) micelles in 20 mM phosphate buffer (pH 8.0). Except for mutant M20R1, the mutants presented decreased mobility upon addition of the membrane mimetic. The arrow denotes the more immobilized population in D17R1 spectrum, which showed up in the presence of the micelles.

**Figure 4:**
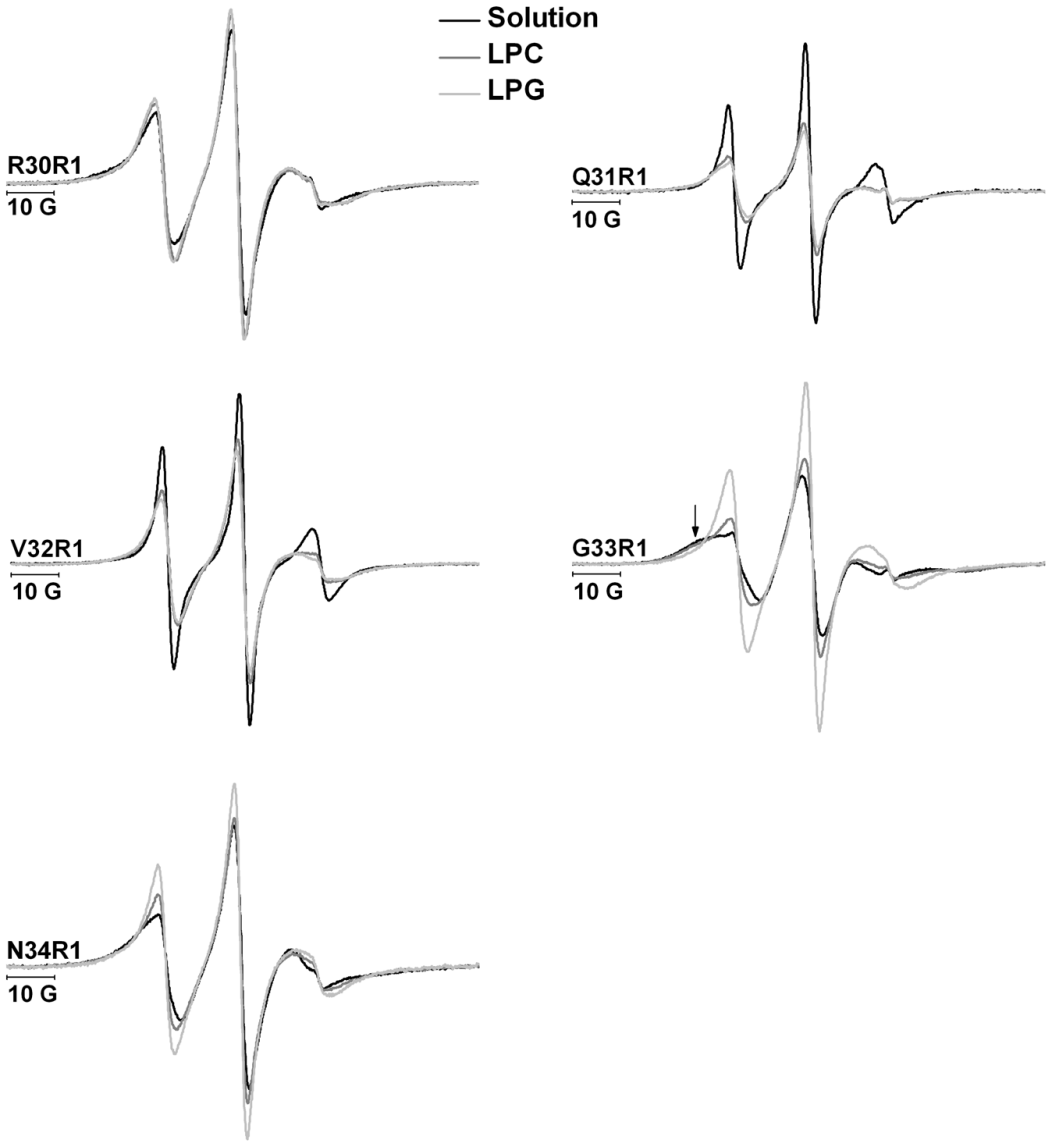
ESR spectra of B-FABP mutants R30R1, Q31R1, V32R1, G33R1 and N34R1 in the absence (black) and in the presence of LPC (gray) or LPG (light gray) micelles in 20 mM phosphate buffer (pH 8.0). The arrow denotes the more immobilized population in the G33R1 spectrum.

Figures 3 and 4 show the ESR spectra of mutants D17R1, E18R1, M20R1 and K21R1 (α-helix A1) and R30R1, Q31R1, V32R1, G33R1 and N34R1 (α-helix A2), respectively. Doubly integrated spectra were normalized to equal areas, which corresponds to the same number of spins, and allows an empirical analysis of the dynamics of MTSSL probe, since spectra with broader lines are less intense, thus reflecting a more restricted mobility of the spin label [59].

The spectral lineshapes in solution reflect the different dynamics experienced by the R1 side chain according to its location along the α-helices. Based on this lineshape analysis the spectra can be divided in two groups.

**Figure 5:**
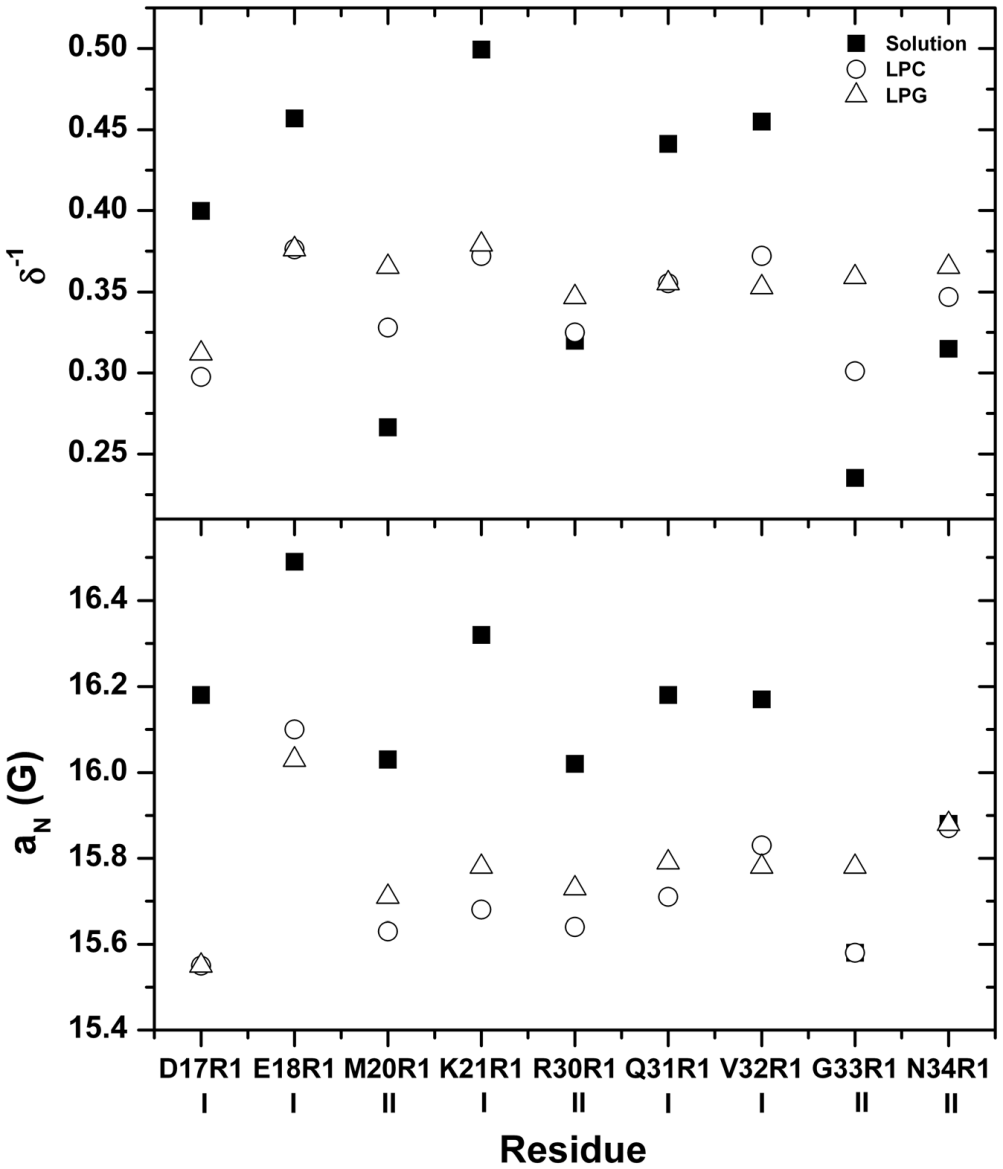
(Top) Calculated parameter δ^−1^ as a measure of backbone dynamics of R1 in solution (black squares) and in the presence of LPC (open circles) or LPG (open triangles) micelles. (Bottom) Calculated values of isotropic hyperfine coupling (a_N_) for the ESR spectra of B-FABP mutants in solution (black squares) and in the presence of LPC (open circles) or LPG (open triangles) micelles. The mutants were divided in two groups (I and II) according to their response to the presence of the membrane model. Group I mutants presented decreased mobility (lower δ^−1^ values) of the R1 side chain in the presence of LPC or LPG micelles. G33R1 was the only mutant showing different responses in terms of a_N_ variations.

The first group (group I) consists of the spectra resulting from the labeling of the residues Asp^17^, Glu^18^, Lys^21^, Gln^31^, and Val^32^ that show narrow lines indicating the high mobility experienced by the R1 side chain when exposed to solvent (black lines in Figures 3 and 4). An empirical analysis of the spectra based on the inverse of the center line width (δ^−1^) [59], which gives estimates of mobility of the side chain R1, and on the isotropic hyperfine splitting (a_N_) [65], which reflects the polarity around the probe, can be employed. As can be seen in Figure 5, the side chain R1 of the above-mentioned mutants have higher values of δ^−1^ and a_N_, consistent with their high degree of mobility and exposure to the aqueous solvent.

In the presence of LPC or LPG micelles, it was observed a significant decrease of mobility of the R1 side chain of those mutants as can seen from the line broadening with a consequent decrease in the amplitude of the spectrum. Decreases in the δ^−1^ and a_N_ values reflect the decreased mobility and polarity, respectively, of the medium surrounding the sidechain R1 in the presence of micelles (LPC or LPG), showing that this side chain is in an environment of lower polarity when compared to the side chain in solution. This indicates that interaction occurs between B-FABP mutants and zwitterionic or negatively charged micelles. The appearance of a second highly immobilized component in the spectra of D17R1 when in the presence of micelles is also note worthy (see arrows in Figure 3).

Group I spectra are associated to residues with side chains that point outwards from the contact region between helices A1 and A2 (see Figure 1) and that, in the presence of micelles, responded with a significant reduction of their side chain dynamics as inferred from the less intense ESR spectra and from the decreased δ^−1^ values. Moreover, a decrease in a_N_ is obtained, which indicates a less polar environment around the spin probe moiety, most likely due to the interaction of B-FABP with the interior of the membrane mimetic. An interesting feature observed only for D17R1 mutant is the coexistence of two spin populations upon addition of micelles, one of which follows the pattern seen for the other mutants (decrease in mobility and polarity). The second population seems to represent a set of spin probes in a very restricted dynamic regime, which might be the result of the stabilization of a very ordered structure in that part of helix A1 in the presence of membranes.

The second set of spectra (group II) corresponds to mutants M20R1, R30R1, G33R1, and N34R1. These spectra indicated lower mobility of the probe in the absence of micelles when compared to the first group (Figures 3 and 4). The side chains are oriented towards the interface between α-helices A1 and A2 in this group of mutants (Figure 1) [12]. The interactions between the side chains present in this region (Met^20^, Val^25^, Gly^26^, Phe^27^ and Arg^30^) are likely the cause of the more restricted motion of MTSSL. In the presence of LPC or LPG micelles, these mutants presented an increase in mobility, as can be noted by the higher amplitude of the spectral lines (Figures 3 and 4) and the increase in the values of the parameter δ^−1^(Figure 5a). This fact can be attributed to conformational changes caused in the α-helices upon binding to micelles, probably leading to an unpacking of the helices (either away from each other or away from the beta sheets) and a consequent increase of the free volume between them, which allowed the side chain R1 to experience higher mobility as indicated by all group II ESR spectra.

As observed for mutants in group I, the spectra of M20R1 and R30R1 showed a decrease in parameter a_N_ (Figure 5b), indicating that the probe is in an environment of lower polarity, probably participating in the interaction with the micelle, and insensible to the charge of lysophospholipids. Interestingly, the a_N_ value for G33R1 increases in the presence of LPG and remained unaltered in LPC, whereas no changes were observed for N34R1. These results suggest that it is the end of α-helix A2 that seems to differentiate between the nature of the membrane models (whether it is charged or not), probably acting as a sensor of lipid charge.

Another interesting feature concerning G33R1is that its spectrum in the absence of micelles reflected the highest degree of immobilization amongst all mutants and it was the only mutant in helix A2 whose spectrum contained two spectral components (see arrows in Figure 4), one of which is highly immobilized and most abundant in the absence of micelles and a more flexible component that becomes dominant upon addition of membranes. This is exactly the opposite situation when compared to the spectra of D17R1, where the two components appeared after mixture with membranes. This interplay of spectral behavior along with the variations of a_N_ values for G33R1 and N34R1 suggest the N-terminal of helix A1 and the C-terminal of helix A2, i.e. the portions farthest away from the loop connecting the helices, have special roles in the mechanism of membrane binding and probably fatty acid delivery.

Residues G33R1 and N34R1, together with residues Leu^23^, Gly^26^, Gln^31^, Val^32^, Thr^36^, Phe^57^, Lys^58^, Asn^59^, Thr^60^ and Ala^75^are located in a structural site shown to be a region that has large fluctuations due to the interaction with B-FABP natural ligands [12], [13], a characteristic that is also shared by I-FABP [40], [66]. Separated by ca.8 Å from residues G33R1 and N34R1, the residues Val^35^ and Phe^57^ may be involved in interactions with the micelle core. In addition, the proximity of residues Lys^37^ and Lys^58^ may be responsible for the observed increased mobility of the side chain of mutant G33R1. The flexibility of this region leads to the interaction of the positive charged side chain of Lys residues with anionic polar heads of LPG micelles and could lead to the increased mobility of R1 side chain due to structural changes in the C-terminal region of the α-helix A2. In the presence of LPC micelles, the interaction leads to a smaller conformational change of α-helix. These results are in agreement with data obtained by Thumser *et al.* [44], which point to a collisional mechanism for FAs delivered by B-FABP. This mechanism is dependent on the phospholipid composition of the acceptor membrane. A 18-fold increase in the throughput of the FA to the acceptor vesicles is observed in the presence of lipids with negatively charged polar head, pointing to an important role for basic residues Lys and Arg in the transient protein-membrane interactions [44]. These basic residues were also considered important in the interaction between biomimetic systems and H-FABP [39] or A-FABP [38]. In several studies involving I- [16], [64], B- [44], H- [39], and A-FABP [38], [67], it was pointed out that the rate of delivery of FAs to zwitterionic membranes is always lower when compared to the negatively charged vesicles, reinforcing the importance of basic residues [32], [38], [39].

The ESR spectra from mutants in helices A1 and A2 indicate that the binding mechanism of B-FABP to membrane models involves conformational changes in those helices that would separate them, thus making enough room to account for the increase in mobility seen in the group II ESR spectra after membrane addition. Group I residues would be structurally stabilized in the membrane interior probably contributing to keep the portal region in an open configuration to allow for the exit of FAs from the protein cavity towards the membrane. Furthermore, it is worth noting the possible role played by the residues located at the beginning and at the end of the portal region (N-terminal of helix A1 and C-terminal of helix A2). In those cases, the ESR spectra showed interplay between residues Asp^17^ and Gly^33^ in terms of the existence of two populations in the presence and absence of membranes, respectively. Gly^33^ (and to some extent Asn^34^ as well) would be more ordered in the absence of membranes, probably contributing to the stabilization of the FA inside the protein. In the presence of membranes and sensing its surface charge (Gly^33^ was the only residue capable of responding differently to LPC or LPG micelles), this portion would undergo a conformational change to facilitate FA release and the opposite region in helix A1 (where Asp^17^ is located) would then get ordered and anchored to the membrane, thus giving rise to a multi-component ESR spectrum.

**Figure 6:**
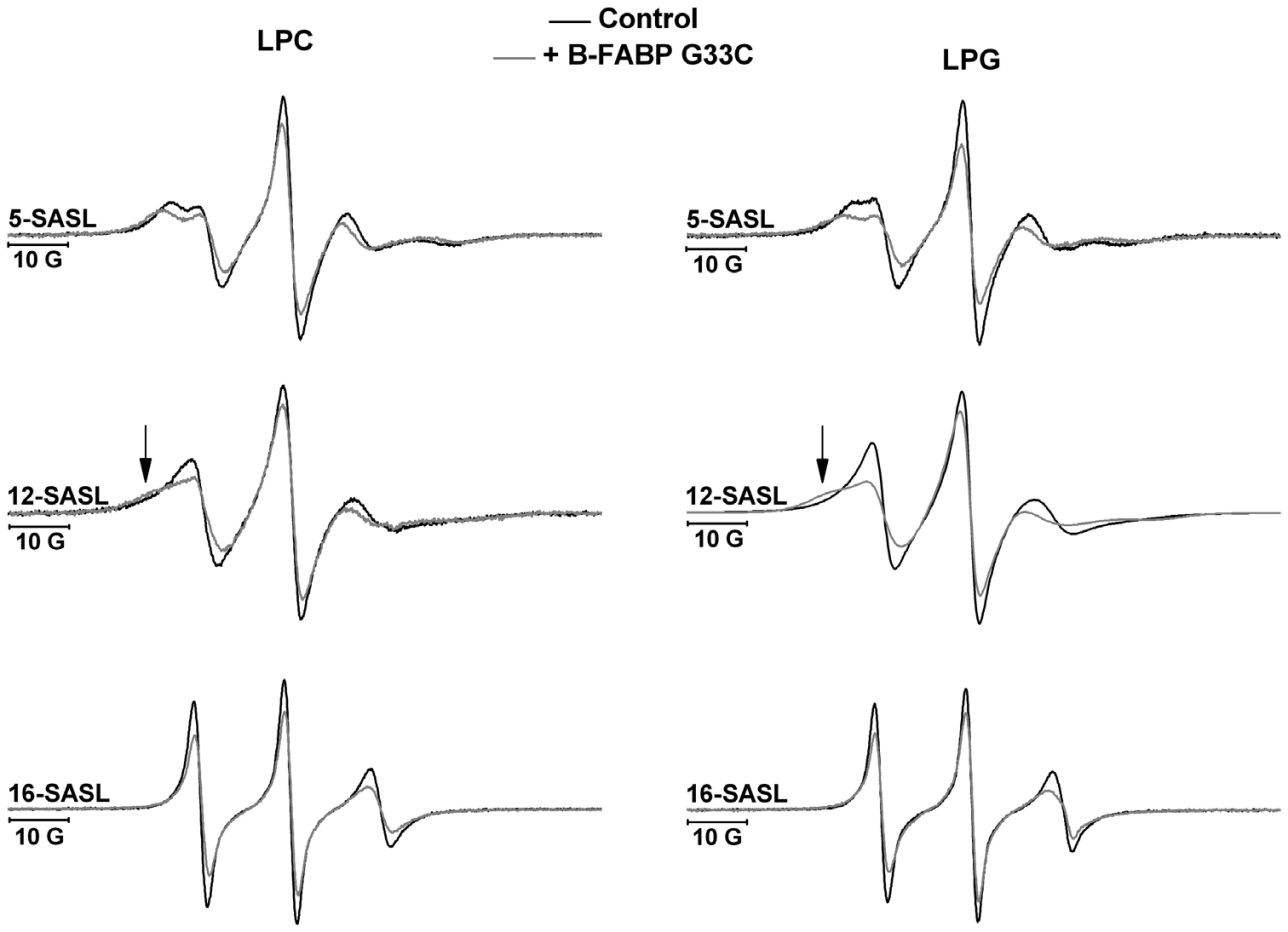
ESR spectra of 5- (top), 12- (middle) and 16-SASL (bottom) incorporated in LPC (left) or LPG (right) micelles. Black lines are the control experiments and gray lines are the spectrum after addition of G33C B-FABP. Changes in lineshape are clearly seen in all cases when mutant G33R1 is added to either LPC or LPG micelles, indicating a less mobile environment around the *n*-SASL probe in the presence of G33R1 B-FABP. The arrow denotes the more immobilized population in the 12-SASL spectrum.

To complement our studies we also looked for changes from the perspective of the LPC and LPG micelles upon B-FABP binding. Measurements were performed with the probes 5-, 12- and 16-SASL incorporated into the membrane model and in the absence and in the presence of the unlabeled mutant B-FABP G33C. This mutant was able to differentiate the nature of the polar heads of lysophospholipids and was chosen for this reason. The probe 5-SASL showed an ESR spectrum corresponding to a higher immobilization in the presence of the protein (top panel in Fig. 6), reflecting an increased order when compared to control. In LPG micelles, an increase of 29% in the order parameter (S) (going from 0.45 to 0.58, see table 1) was observed in the presence of B-FABP G33C when compared to the control. On the other hand, in LPC micelles under the same conditions, the increase was 19% (from 0.51 to 0.60, see Table 1). These results reinforce the importance of electrostatic interactions between the basic residues on the surface of the protein and the negatively charged polar head of LPG micelles, confirming the data obtained for the labeled protein. The increased order of the biomimetic system in the presence of ligands is reported in the literature, being observed in bicelles in the presence of lanthanide ions [72], in DMPC and DTPG vesicles in the presence of biologically active peptide melittin [73], in vesicles of phosphatidylcholine and sphingomyelin in the presence of StI and StII toxins from sea anemone [74] and SDS or HPS micelles in the presence of local anesthetic tetracaine [75].

**Table 1:** Parameters determined from the ESR spectra of *n*-SASL spin label incorporated in the membrane mimetic (LPC or LPG) as discussed in Materials and Methods.

Spin label	Parameter	LPC		LPG	
		Control	+ B-FABP G33C	Control	+ B-FABP G33C
**5-SASL**	**S**	0.51	0.60	0.45	0.58
**12-SASL**	**a_N_ (G)**	14.95	14.75	14.68	14.48
**16-SASL**	**τ_C_ (ns)**	1.12	1.48	0.96	1.48
	**a_N_ (G)**	15.14	15.05	15.29	15.29

As for the probe 12-SASL, its ESR spectra in the presence of LPC or LPG micelles showed broader lines when compared to the control (mid panel in Fig. 6). Broader lines are characteristic of populations that experience a lower mobility. Furthermore, a second spectral component was observed, characteristic of a highly immobilized population (see arrows in the mid panel of Fig. 6). Upon protein binding, the spin labeled FAs are divided in two populations. The first one corresponds to probe molecules that are in the bulk of membrane and give rise to ESR signals similar to the control experiment. The second population is attributed to probe molecules in the vicinity of the protein and that have thus their motion restricted by the presence of B-FABP. This is what is known as boundary lipid, in this case boundary probe, and has been observed before in lipid-protein interactions [76]–[79].These data are consistent with the results for 5-SASL, indicating that the protein binds to micelles and induces an ordering effect in the molecules that are in close proximity. The isotropic hyperfine splitting (a_N_) for 12-SASL incorporated in both micelles is slightly lower when compared to the control (Table 1), indicating a microenvironment of lower polarity in the presence of B-FABP G33C. This may point to a higher packing of the micelle, also consistent with the increase of the 5-SASL order parameter.

Similar behavior was observed for the 16-SASL probe, where the reduction of dynamics experienced by the nitroxide moiety was reflected in the broadening of the spectral lines (bottom panel in Fig. 6). 16-SASL incorporated in LPG micelles and in the presence of B-FABP G33C had its rotational correlation time (τ_C_) increased by 54% compared to the same micelle in the absence of protein (Table 1). Under identical conditions, 16-SASL incorporated in LPC micelles had its τ_C_ increased by 32% (Table 1). Once more our data point to a larger alteration in LPG than in LPC micelles, suggesting a stronger interaction between the B-FABP G33C and negatively charged micelles. No significant variations were found in the values of a_N_ (Table 1), and this can be explained by the fact that the 16-SASL probe is located at the end of the fatty acid acyl chain, within the core of the micelle, where no significant disturbances could be detected in medium polarity.

The binding of collisional B-FABP to the target membrane is certainly mediated by electrostatic interactions with contributions from the surface potential of the protein and of the membrane as observed for other peripheral binding proteins [68]–[71]. Previous studies on FABP [32], [38], [39] showed that point mutations mainly affect the kinetics of delivery of FA to the membrane, without changing so much the mechanism of binding. In a very interesting study on A-FABP [38], the mechanism of delivery was changed from collisional to diffusional by acetylation of all lysine residues on the surface of the protein, which clearly indicates the relevance of those residues in determining the mode of delivery. Herr *et al.* [39] also showed that mutation of the Lys^22^ makes the protein insensitive to the membrane charge. Our results suggest that a similar situation happens with B-FABP since mutant K21R1, where the Lys^21^ residue is absent, does not seem to distinguish between LPC or LPG micelles (Figure 3), whereas mutants M20R1 (Figure 3), G33R1 and N34R1 (Figure 4) do. Moreover, our results indicate that in the absence of some charged residues (such as in mutants D17R1, E18R1, K21R1 and R30R1, Figures 3 and 4), the protein still interacts with the membrane mimetic but is not able to differentiate its charge. This indicates that there is not a single residue responsible for the collisional delivery since the protein keeps interacting with the membrane when one of those charged residues is removed.

Molecular dynamics studies have shown the importance of the electrostatic surface potential for the FABP-membrane interaction, such as A-FABP [41] and I-FABP [80]. Zamarreño *et al.* [80] showed that there is not a key residue for the protein-membrane interaction, but the contribution of a surface potential instead. Our data also point to the relevance of the surface potential, since point mutations of acidic (Asp^17^ and Glu^18^) or basic (Lys^21^ and Arg^30^) residues on the helices do not seem to interfere with the protein ability to bind to micelles, thus indicating that is the whole surface potential of the contact portion of the protein structure that is relevant to support the interaction. As reported in the literature [68]–[70], even residues that have a negative charge on their side chain may be responsible for interaction with anionic membranes, since in the vicinity of the negative headgroup there is a higher concentration of H^+^, which decreases the pH in this region and can cause the protonation of the acidic side chain residues. This can decrease the repulsion between acidic side chains and negative headgroups. Furthermore, the data obtained with the probes 5-, 12- and 16-SASL incorporated in LPC or LPG micelles showed that the B-FABP induced alterations in both cases but to different extents, with the larger changes observed for LPG membranes, which may reflect the preference of the protein for negatively charged micelles, supporting the idea that B-FABP performs the FA delivery through collisional mechanism to acceptor membranes [44].

Taking these results together, we can say that B-FABP interacts with both zwitterionic and negatively charged micelles through the portal region, as already observed for other FABPs [40], [62], [66], [81]. Our data indicate that, from the perspective of the protein, the N-terminal of helix A1 and the C-terminal of helix A2 play a unique role in the process of membrane recognition and binding, containing residues that would be responsible for the discrimination of the charge of the lipid polar head that form the acceptor vesicle. Moreover, residues that have their side chains oriented in between the two helices (ESR spectra in group II) undergo a transition from a more to a less packed (or with less restricted dynamics) conformation upon membrane binding. On the other hand, residues whose side chains point outwards, facing the solvent in solution (ESR spectra in group I), experienced an opposite effect and are in an environment of more restriction of motion in the presence of micelles. This is in perfect agreement with the description of such interaction gained by using the fatty acid spin probes incorporated in the micelles. For all *n*-SASL probes it was observed an increase in packing (or a decrease in mobility) when B-FABP and micelles are brought together. Then, this tighter packing induced by the protein in the close-by regions of the micelles is also seen in the protein residues of group I as the decrease of mobility described above. Overall, the protein stabilizes the outside parts of helices A1 and A2 by docking them to the membrane model and adopting a more opened conformation to increase the free space between the helices, which may facilitate the delivery of the FA from the protein binding site to the membrane.

Thus, based on the model proposed by Corsico *et al.* [40] for the FA delivery by I-FABP, we suggest a similar mechanism for B-FABP, where we would have: (i) an initial interaction between the protein and the acceptor membrane, shown in our results as a decrease in the mobility of R1 side chain in residues exposed to solvent (D17R1, E18R1, K21R1, Q31R1 and V32R1) as well as an increase in packing of LPC or LPG micelles, and (ii) conformational changes in both α-helices, from a more ordered state (in solution) to a less ordered state (membrane bound), as shown by the spectra of the mutants M20R1, R30R1, G33R1 and N34R1, resulting in delivery of the fatty acid to the acceptor membrane. The greater disordering perceived by G33R1 mutant in the presence of LPG micelles could in fact be triggering the highest rates of fatty acids transfer to negatively charged membranes found in literature for B-FABP [44]. This reasoning could be applied to residue Ala^31^ in I-FABP [16], [64], residue Ala^33^ in H-FABP [39], and residue Ala^41^ in A-FABP [38], [67], all situated at the C-terminal of α-helix A2. To the best of our knowledge these data represent a detailed description and the first direct indication of the interaction B-FABP-membrane, since previous data [44] only showed indirect evidences of this interaction.

## References

[1] Bernlohr DA, Simpson MA, Hertzel AV, Banaszak LJ (1997) Intracellular lipid-binding proteins and their genes. Annu Rev Nutr 17: 277–303. 9240929 10.1146/annurev.nutr.17.1.277

[2] Hotamisligil GS (2006) Inflammation and metabolic disorders. Nature 444: 860–867. 17167474 10.1038/nature05485

[3] Saltiel AR, Kahn CR (2001) Insulin signalling and the regulation of glucose and lipid metabolism. Nature 414: 799–806. 11742412 10.1038/414799a

[4] Funk CD (2001) Prostaglandins and leukotrienes: advances in eicosanoid biology. Science 294: 1871–1875. 11729303 10.1126/science.294.5548.1871

[5] Serhan CN (2007) Resolution phase of inflammation: novel endogenous anti-inflammatory and proresolving lipid mediators and pathways. Annu Rev Immunol 25: 101–137. 17090225 10.1146/annurev.immunol.25.022106.141647

[6] Schachtrup C, Emmler T, Bleck B, Sandqvist A, Spener F (2004) Functional analysis of peroxisome-proliferator-responsive element motifs in genes of fatty acid-binding proteins. Biochem J 382: 239–245. 15130092 10.1042/BJ20031340 PMC1133936

[7] Motojima K (2000) Differential effects of PPAR alpha activators on induction of ectopic expression of tissue-specific fatty acid binding protein genes in the mouse liver. Int J Biochem Cell Biol 32: 1085–1092. 11091141 10.1016/s1357-2725(00)00046-7

[8] Tan NS, Shaw NS, Vinckenbosch N, Liu P, Yasmin R, et al (2002) Selective cooperation between fatty acid binding proteins and peroxisome proliferator-activated receptors in regulating transcription. Mol Cell Biol 22: 5114–5127. 12077340 10.1128/MCB.22.14.5114-5127.2002 PMC139777

[9] Wolfrum C, Borrmann CM, Borchers T, Spener F (2001) Fatty acids and hypolipidemic drugs regulate peroxisome proliferator-activated receptors alpha - and gamma-mediated gene expression via liver fatty acid binding protein: a signaling path to the nucleus. Proc Natl Acad Sci USA 98: 2323–2328. 11226238 10.1073/pnas.051619898 PMC30137

[10] Furuhashi M, Hotamisligil GS (2008) Fatty acid-binding proteins: role in metabolic diseases and potential as drug targets. Nat Rev Drug Discov 7: 489–503. 18511927 10.1038/nrd2589 PMC2821027

[11] Hohoff C, Spener F (1998) Fatty acid binding proteins and mammary-derived growth inhibitor. Fett-Lipid 100: 252–263.

[12] Rademacher M, Zimmerman AW, Ruterjans H, Veerkamp JH, Lucke C (2002) Solution structure of fatty acid-binding protein from human brain. Mol Cell Biochem 239: 61–68. 12479569

[13] Balendiran GK, Schnutgen F, Scapin G, Borchers T, Xhong N, et al (2000) Crystal structure and thermodynamic analysis of human brain fatty acid-binding protein. J Biol Chem 275: 27045–27054. 10854433 10.1074/jbc.M003001200

[14] Sacchettini JC, Gordon JI, Banaszak LJ (1989) Crystal structure of rat intestinal fatty acid-binding protein J Mol Biol. 208: 327–339. 10.1016/0022-2836(89)90392-6 2671390

[15] Xu ZH, Bernlohr DA, Banaszak LJ (1992) Crystal structure of recombinant murine adipocyte lipid-binding protein. Biochemistry 31: 3484–3492. 1554730 10.1021/bi00128a024

[16] Franchini GR, Storch J, Corsico B (2008) The integrity of the alpha-helical domain of intestinal fatty acid binding protein is essential for the collision-mediated transfer of fatty acids to phospholipid membranes. Biochim Biophys Acta 1781: 192–199. 18284926 10.1016/j.bbalip.2008.01.005 PMC4319566

[17] Shimizu F, Watanabe TK, Shinomiya H, Nakamura Y, Fujiwara T (1997) Isolation and expression of a cDNA for human brain fatty acid-binding protein (B-FABP). Biochim Biophys Acta 1354: 24–28. 9375786 10.1016/s0167-4781(97)00115-2

[18] Bourre JM, Francois M, Youyou A, Dumont O, Piciotti M, et al (1989) The effects of dietary alpha-linolenic acid on the composition of nerve membranes, enzymatic-activity, amplitude of electrophysiological parameters, resistance to poisons and performance of learning-tasks in rats. J Nutr 119: 1880–1892. 2576038 10.1093/jn/119.12.1880

[19] Innis SM, Sprecher H, Hachey D, Edmond J, Anderson RE (1999) Neonatal polyunsaturated fatty acid metabolism. Lipids 34: 139–149. 10102240 10.1007/s11745-999-0348-x

[20] Connor WE, Neuringer M, Barstad L, Lin DS (1984) Dietary deprivation of linolenic acid in Rhesus-monkeys - effects on plasma and tissue fatty-acid composition and on visual function. Trans Assoc Am Physicians 97: 1–9. 6535333

[21] Yamamoto N, Saitoh M, Moriuchi A, Nomura M, Okuyama H (1987) Effect of dietary alpha-linolenate-linoleate balance on brain lipid compositions and learning-ability of rats. J Lipid Res 28: 144–151. 2883248

[22] Feng L, Hatten ME, Heintz N (1994) Brain lipid-binding protein (BLBP) - a novel signaling system in the developing mammalian CNS. Neuron 12: 895–908. 8161459 10.1016/0896-6273(94)90341-7

[23] Sanchez-Font MF, Bosch-Comas A, Gonzalez-Duarte R, Marfany G (2003) Overexpression of FABP7 in Down syndrome fetal brains is associated with PKNOX1 gene-dosage imbalance. Nucleic Acids Res 31: 2769–2777. 12771203 10.1093/nar/gkg396 PMC156729

[24] Watanabe A, Toyota T, Owada Y, Hayashi T, Iwayama Y, et al (2007) Fabp7 maps to a quantitative trait locus for a schizophrenia endophenotype. PloS Biol 5: 2469–2483. 10.1371/journal.pbio.0050297 PMC2071943 18001149

[25] Owada Y, Abdelwahab SA, Kitanaka N, Sakagami H, Takano H, et al (2006) Altered emotional behavioral responses in mice lacking brain-type fatty acid-binding protein gene. Eur J Neurosci 24: 175–187. 16882015 10.1111/j.1460-9568.2006.04855.x

[26] Shi YE, Ni J, Xiao GW, Liu YE, Fuchs A, et al (1997) Antitumor activity of the novel human breast cancer growth inhibitor, mammary-derived growth inhibitor-related gene, MRG. Cancer Res 57: 3084–3091. 9242429

[27] Hohoff C, Spener F (1998) Correspondence re: Y.E. Shi et al, Antitumor activity of the novel human breast cancer growth inhibitor, mammary-derived growth inhibitor-related gene, MRG, Cancer Res, 57: 3084–3091,1997. Cancer Res 58: 4015–4016. 9731516

[28] Takaoka N, Takayama T, Teratani T, Sugiyama T, Mugiya S, et al (2011) Analysis of the regulation of fatty acid binding protein 7 expression in human renal carcinoma cell lines. BMC Mol Biol 12: 31. 21771320 10.1186/1471-2199-12-31 PMC3162894

[29] Retrosi G, Sebire NJ, Bishay M, Kiely EM, Anderson J, et al (2011) Brain lipid–binding protein: a marker of differentiation in neuroblastic tumors. J Pediatr Surg 46: 1197–1200. 21683222 10.1016/j.jpedsurg.2011.03.053

[30] Tolle A, Krause H, Miller K, Jung K, Stephan C (2011) Importance of brain-type fatty acid binding protein for cell-biological processes in human renal carcinoma cells. Oncol Rep 25: 1307–1312. 21399875 10.3892/or.2011.1209

[31] Mita R, Beaulieu MJ, Field C, Godbout R (2010) Brain fatty acid-binding protein and omega-3/omega-6 fatty acids: mechanistic insight into malignant glioma cell migration. J Biol Chem 285: 37005–37015. 20834042 10.1074/jbc.M110.170076 PMC2978629

[32] Falomir-Lockhart LJ, Laborde L, Kahn PC, Storch J, Corsico B (2006) Protein-membrane interaction and fatty acid transfer from intestinal fatty acid-binding protein to membranes. Support for a multistep process. J Biol Chem 281: 13979–13989. 16551626 10.1074/jbc.M511943200

[33] Thumser AEA, Storch J (2000) Liver and intestinal fatty acid-binding proteins obtain fatty acids from phospholipd membranes by different mechanisms. J Lipid Res 41: 647–656. 10744786

[34] Hagan RM, Worner-Gibbs J, Wilton DC (2008) The interaction of liver fatty-acid-binding protein (FABP) with anionic phospholipid vesicles: is there extended phospholipid anchorage under these conditions? Biochem J 410: 123–129. 17935485 10.1042/BJ20071109

[35] Davies JK, Thumser AEA, Wilton DC (1999) Binding of recombinant rat liver fatty acid-binding protein to small anionic phospholipid vesicles results in ligand release: a model for interfacial binding and fatty acid targeting. Biochemistry 38: 16932–16940. 10606528 10.1021/bi991926q

[36] Davies JK, Hagan RM, Wilton DC (2002) Effect of charge reversal mutations on the ligand- and membrane-binding properties of liver fatty acid-binding protein. J Biol Chem 277: 48395–48402. 12379651 10.1074/jbc.M208141200

[37] Storch J, Thumser AEA (2000) The fatty acid transport function of fatty acid-binding proteins. Biochim Biophys Acta 1486: 28–44. 10856711 10.1016/s1388-1981(00)00046-9

[38] Herr FM, Matarese V, Bernlohr DA, Storch J (1995) Surface lysine residues modulate the colllisional transfer of fatty acid from adipocyte fatty acid binding protein to membranes. Biochemistry 34: 11840–11845. 7547918 10.1021/bi00037a023

[39] Herr FM, Aronson J, Storch J (1996) Role of portal region lysine residues in electrostatic interactions between heart fatty acid binding protein and phospholipid membranes. Biochemistry 35: 1296–1303. 8573586 10.1021/bi952204b

[40] Corsico B, Cistola DP, Frieden C, Storch J (1998) The helical domais of intestinal fatty acid binding protein is critical for collisional transfer to phospholipid membranes. Proc Natl Acad Sci U S A 95: 12174–12178. 9770459 10.1073/pnas.95.21.12174 PMC22804

[41] LiCata VJ, Bernlohr DA (1998) Surface properties of adipocyte lipid-binding protein: response to lipid binding, and comparison with homologous proteins. Proteins 33: 577–589. 9849941 10.1002/(sici)1097-0134(19981201)33:4<577::aid-prot10>3.0.co;2-2

[42] Kim HK, Storch J (1992) Mechanism of free fatty acid transfer from rat heart fatty acid-binding protein to phospholipid membranes J Biol Chem. 267: 20051–20056. 1400322

[43] Hsu KT, Storch J (1996) Fatty acid transfer from liver and intestinal fatty acid-binding proteins to membranes occurs by different mechanisms. J Biol Chem 271: 13317–13323. 8662836 10.1074/jbc.271.23.13317

[44] Thumser AEA, Tsai J, Storch J (2001) Collision-mediated transfer of long-chain fatty acids by neural tissue fatty acid-binding proteins (FABP). J Mol Neurosci 16: 143–150. 11478369 10.1385/JMN:16:2-3:143

[45] McHaourab HS, Lietzow MA, Hideg K, Hubbell WL (1996) Motion of spin-labeled side chains in T4 lysozyme, correlation with protein structure and dynamics. Biochemistry 35: 7692–7704. 8672470 10.1021/bi960482k

[46] Oh KJ, Zhan HJ, Cui C, Hideg K, Collier RJ, et al (1996) Organization of diphtheria toxin T domain in bilayers: a site-directed spin labeling study. Science 273: 810–812. 8670424 10.1126/science.273.5276.810

[47] Perozo E, Cortes DM, Cuello LG (1998) Three-dimensional architecture and gating mechanism of a K+ channel studied by EPR spectroscopy. Nat Struct Biol 5: 459–469. 9628484 10.1038/nsb0698-459

[48] Klug CS, Su WY, Feix JB (1997) Mapping of the residues involved in a proposed beta-strand located in the ferric enterobactin receptor FepA using site-directed spin-labeling. Biochemistry 36: 13027–13033. 9335564 10.1021/bi971232m

[49] Lin Y (1998) Docking phospholipase A2 on membranes using electrostatic potential-modulated spin relaxation magnetic resonance. Science 279: 1925–1929. 9506941 10.1126/science.279.5358.1925 PMC3443684

[50] McHaourab HS, Oh KJ, Fang CJ, Hubbell WL (1997) Conformation of T4 lysozyme in solution. Hinge-bending motion and the substrate-induced conformational transition studied by site-directed spin labeling. Biochemistry 36: 307–316. 9003182 10.1021/bi962114m

[51] Couto SG, Cristina Nonato M, Costa-Filho AJ (2011) Site directed spin labeling studies of Escherichia coli dihydroorotate dehydrogenase N-terminal extension. Biochem Biophys Res Commun. 10.1016/j.bbrc.2011.09.092 21986535

[52] Addona GH, Andrews SH, Cafiso DS (1997) Estimating the electrostatic potential at the acetylcholine receptor agonist site using power saturation EPR. Biochim Biophys Acta 1329: 74–84. 9370246 10.1016/s0005-2736(97)00089-8

[53] McConnell HM, Hubbell WL (1971) Molecular motion in spin-labeled phospholipids and membranes. J Am Chem Soc 93: 314–326. 5541516 10.1021/ja00731a005

[54] Berliner LJ (1979) Spin Labeling: Theory and Applications; Berliner LJ, editor. New York: Academic Press Inc.

[55] Hubbell WL, McHaourab HS, Altenbach C, Lietzow MA (1996) Watching proteins move using site-directed spin labeling. Structure 4: 779–783. 8805569 10.1016/s0969-2126(96)00085-8

[56] Borbat PP, Costa-Filho AJ, Earle KA, Moscicki JK, Freed JH (2001) Electron spin resonance in studies of membranes and proteins. Science 291: 266–269. 11253218 10.1126/science.291.5502.266

[57] Zimmerman AW, Rademacher M, Ruterjans H, Lucke C, Veerkamp JH (1999) Functional and conformational characterization of new mutants of heart fatty acid-binding protein. Biochem J 344: 495–501. 10567233 PMC1220668

[58] Oeemig JS, Jorgensen ML, Hansen MS, Petersen EI, Duroux L, et al (2009) Backbone and sidechain 1H, 13C and 15N resonance assignments of the human brain-type fatty acid binding protein (FABP7) in its apo form and the holo forms binding to DHA, oleic acid, linoleic acid and elaidic acid. Biomol NMR Assign 3: 89–93. 19636954 10.1007/s12104-009-9148-6

[59] Columbus L, Hubbell WL (2004) Mapping backbone dynamics in solution with site-directed spin labeling: GCN4-58 bZip free and bound to DNA. Biochemistry 43: 7273–7287. 15182173 10.1021/bi0497906

[60] Schreier S, Polnaszek CF, Smith ICP (1978) Spin labels in membranes. Problems in practice. Biochim Biophys Acta 515: 375–436. 10.1016/0304-4157(78)90011-4 215206

[61] Janes RW, Wallace BA (2009) An introduction to Circular Dichroism and Synchrotron Radiation Circular Dichroism spectroscopy. In: Wallace BA, Janes RW, editors. Modern techniques for Circular Dichroism and Synchrotron Radiation Circular Dichroism spectroscopy. Amsterdam, Netherlands: IOS Press. pp. 1–18.

[62] Corsico B, Liou HL, Storch J (2004) The alpha-helical domail of liver fatty acid binding protein is responsible for the diffusion-mediated transfer of fatty acids to phospholipid membranes. Biochemistry 43: 3600–3607. 15035630 10.1021/bi0357356

[63] Kelly SM, Jess TJ, Price NC (2005) How to study proteins by circular dichroism. Biochim Biophys Acta 1751: 119–139. 16027053 10.1016/j.bbapap.2005.06.005

[64] Corsico B, Franchini GR, Hsu KT, Storch J (2005) Fatty acid transfer from intestinal fatty acid binding protein to membranes: electrostatic and hydrophobic interactions. J Lipid Res 46: 1765–1772. 15863832 10.1194/jlr.M500140-JLR200

[65] Schreier S, Frezzatti WA, Araujo PS, Chaimovich H, Cuccovia IM (1984) Effect of lipid membranes on the apparent pK of the local anesthetic tetracaine. Spin label and titration studies. Biochim Biophys Acta 769: 231–237. 6318824 10.1016/0005-2736(84)90027-0

[66] Hodsdon ME, Cistola DP (1997) Discrete backbone disorder in the nuclear magnetic resonance structure of apo intestinal fatty acid-binding protein: implications for the mechanism of ligand entry. Biochemistry 36: 1450–1460. 9063893 10.1021/bi961890r

[67] Liou HL, Storch J (2001) Role of surface lysine residues of adipocyte fatty acid-binding protein in fatty acid transfer to phospholipid vesicles. Biochemistry 40: 6475–6485. 11371211 10.1021/bi0101042

[68] van der Goot FG, Gonzalez-Manas JM, Lakey JH, Pattus F (1991) A ‘molten-globule’ membrane-insertion intermediate of the pore-forming domain of colicin A. Nature. 354: 408–410. 10.1038/354408a0 1956406

[69] Dunne SJ, Cornell RB, Johnson JE, Glover NR, Tracey AS (1996) Structure of the membrane binding domain of CTP: phosphocholine cytidylyltransferase. Biochemistry 35: 11975–11984. 8810902 10.1021/bi960821+

[70] Johnson JE, Xie M, Singh LM, Edge R, Cornell RB (2003) Both acidic and basic amino acids in an amphitropic enzyme, CTP:phosphocholine cytidylyltransferase, dictate its selectivity for anionic membranes. J Biol Chem 278: 514–522. 12401806 10.1074/jbc.M206072200

[71] Bustad HJ, Skjaerven L, Ying M, Halskau O, Baumann A, et al (2012) The peripheral binding of 14-3-3gamma to membranes involves isoform-specific histidine residues. PLoS One 7: e49671. 23189152 10.1371/journal.pone.0049671 PMC3506662

[72] Inbaraj JJ, Nusair NA, Lorigan GA (2004) Investigating magnetically aligned phospholipid bilayers with EPR spectroscopy at Q-band (35 GHz): optimization and comparison with X-band (9 GHz). J Magn Reson 171: 71–79. 15504684 10.1016/j.jmr.2004.08.002

[73] Kleinschmidt JH, Mahaney JE, Thomas DD, Marsh D (1997) Interaction of bee venom melittin with zwitterionic and negatively charged phospholipid bilayers: a spin-label electron spin resonance study. Biophys J 72: 767–778. 9017202 10.1016/s0006-3495(97)78711-3 PMC1185600

[74] Alvarez C, Casallanovo F, Shida CS, Nogueira LV, Martinez D, et al (2003) Binding of sea anemone pore-forming toxins sticholysins I and II to interfaces - Modulation of conformation and activity, and lipid–protein interaction. Chem Phys Lipids 122: 97–105. 12598041 10.1016/s0009-3084(02)00181-0

[75] Teixeira CV, Itri R, Casallanovo F, Schreier S (2001) Local anesthetic-induced microscopic and mesoscopic effects in micelles. A fluorescence, spin label and SAXS study. Biochim Biophys Acta 1510: 93–105. 11342150 10.1016/s0005-2736(00)00338-2

[76] Jost PC, Griffith OH, Capaldi RA, Vanderkooi G (1973) Evidence for boundary lipid in membranes. Proc Natl Acad Sci U S A 70: 480–484. 4346892 10.1073/pnas.70.2.480 PMC433287

[77] Kang SY, Gutowsky HS, Hsung JC, Jacobs R, King TE, et al (1979) Nuclear magnetic resonance investigation of the cytochrome oxidase-phospholipid interaction: a new model for boundary lipid. Biochemistry 18: 3257–3267. 223629 10.1021/bi00582a010

[78] Costa-Filho AJ, Crepeau RH, Borbat PP, Ge M, Freed JH (2003) Lipid-Gramicidn interactions: dynamic structure of the boundary lipid by 2D-ELDOR. Biophys J 84: 3364–3378. 12719265 10.1016/S0006-3495(03)70060-5 PMC1302896

[79] Couto SG, Nonato MC, Costa-Filho AJ (2008) Defects in vesicle core induced by Escherichia coli dihydroorotate dehydrogenase. Biophys J 94: 1746–1753. 17993483 10.1529/biophysj.107.120055 PMC2242746

[80] Zamarreno F, Herrera FE, Corsico B, Costabel MD (2012) Similar structures but different mechanisms: Prediction of FABPs-membrane interaction by electrostatic calculation. Biochim Biophys Acta 1818: 1691–1697. 22446190 10.1016/j.bbamem.2012.03.003

[81] Mihajlovic M, Lazaridis T (2007) Modeling fatty acid delivery from intestinal fatty acid binding protein to a membrane. Protein Sci 16: 2042–2055. 17660261 10.1110/ps.072875307 PMC2206986

